# Towards User-Friendly Spelling with an Auditory Brain-Computer Interface: The CharStreamer Paradigm

**DOI:** 10.1371/journal.pone.0098322

**Published:** 2014-06-02

**Authors:** Johannes Höhne, Michael Tangermann

**Affiliations:** 1 Machine Learning Laboratory, Berlin Institute of Technology, Berlin, Germany; 2 Neurotechnology group, Berlin Institute of Technology, Berlin, Germany; 3 BrainLinks-BrainTools Excellence Cluster, University of Freiburg, Freiburg, Germany; University of Adelaide, Australia

## Abstract

Realizing the decoding of brain signals into control commands, brain-computer interfaces (BCI) aim to establish an alternative communication pathway for locked-in patients. In contrast to most visual BCI approaches which use event-related potentials (ERP) of the electroencephalogram, auditory BCI systems are challenged with ERP responses, which are less class-discriminant between attended and unattended stimuli. Furthermore, these auditory approaches have more complex interfaces which imposes a substantial workload on their users. Aiming for a maximally user-friendly spelling interface, this study introduces a novel auditory paradigm: “CharStreamer”. The speller can be used with an instruction as simple as “please attend to what you want to spell”. The stimuli of CharStreamer comprise 30 spoken sounds of letters and actions. As each of them is represented by the sound of itself and not by an artificial substitute, it can be selected in a one-step procedure. The mental mapping effort (sound stimuli to actions) is thus minimized. Usability is further accounted for by an alphabetical stimulus presentation: contrary to random presentation orders, the user can foresee the presentation time of the target letter sound. Healthy, normal hearing users (n = 10) of the CharStreamer paradigm displayed ERP responses that systematically differed between target and non-target sounds. Class-discriminant features, however, varied individually from the typical N1-P2 complex and P3 ERP components found in control conditions with random sequences. To fully exploit the sequential presentation structure of CharStreamer, novel data analysis approaches and classification methods were introduced. The results of online spelling tests showed that a competitive spelling speed can be achieved with CharStreamer. With respect to user rating, it clearly outperforms a control setup with random presentation sequences.

## Introduction

Communication is among the most basic and most urgent needs of human beings. Thus assistive technology devices are applied in case normal communication pathways are impaired. Among them, brain-computer interfaces (BCIs) aim to establish communication channels which are independent of muscle movements. BCI systems exploit brain signals like the non-invasive electroencephalogram (EEG) using real-time data analysis methods in order to decode user intentions and control commands. In addition to prevailing BCI concepts, which make use of visual event-related potentials (ERPs) and self-driven imagery tasks, recent studies proposed tactile and auditory paradigms to broaden the applicability of BCI (for further discussion, see [1]–[8]). However, such paradigms which are independent of the visual pathway tend to be more complex and less intuitive to use compared to their visual counterparts. This becomes obvious, when comparing existing auditory (or generally non-visual) BCI paradigms to the most frequently used and probably the most successful visual BCI paradigm, the MatrixSpeller [9]: to operate it, users do not require instructions beyond the hint to mentally focus on the desired symbol. All available symbols are present on the screen at all times. As the paradigm is following the concept of “what you see is what you get”, only low workload is imposed onto the user to select a symbol. If users are capable of directing their gaze to the desired symbol and keep it there, the symbols of the entire alphabet are reachable within one logical selection step. Thus, there is a one-to-one mapping from stimuli to the intended action, which is very intuitive.

Existing non-visual spelling paradigms are far from such simple concepts. Their control only has a low degree of freedom and an intrinsically lower communication bandwidth. Thus, the complex options offered in most real-world situations (or a spelling task) can not be controlled directly. For this reason, a user interface of BCI communication software typically needs to restrict the number of possible control actions at each step to a small, but feasible set. As a result, the selection of a symbol requires the execution of a series of control steps. Determining a suitable mapping from (few) BCI control options to the (high) complexity of an application is a critical design decision which has been approached in many different ways [10]–[12].

The mapping introduces an extra level of vicariousness, which bears a number of difficulties in terms of usability. Firstly, sub-steps to reach a goal (e.g. along trees, into the depth of menus etc.) conflict with imperfect control signals as errors accumulate. Secondly, a spelling tree either needs to be memorized or presented constantly to the user. Thirdly, the user needs to cope with a large cognitive distance between low-level control actions (e.g. selecting the third class) and high-level goals (e.g. spelling “M”). Obviously those three aspects can introduce a non-neglectable extra workload for the BCI user. Although these kind of mappings have been optimized in various ways for spelling applications [13]–[15], the resulting interfaces are far more complex than the logical one-step procedure of the visual MatrixSpeller and in the RSVP paradigm [16]. In the latter paradigm, however, the user needs to at least memorize the desired symbol during the full duration of the selection step, which may comprise tens of seconds of stimulus presentation. While healthy study participants in good condition may be able to use such “indirect” interfaces despite of the enhanced workload requirements, it remains a problem and a high entrance barrier for many patients [17], [18]. But also for healthy persons, it severely limits the usability of the application [19].

This observation motivates a novel auditory BCI approach which is introduced in the presented study. The “CharStreamer” paradigm was designed in order to eliminate the above-mentioned mapping problems. Aiming for a simple-to-use auditory paradigm, the CharStreamer strives to realize two main goals:

1. Every symbol can be selected within a single step.
2. Every symbol is represented by the sound of itself, not by an artificial substitute.

Moreover, a third aspect of complexity was challenged. Typically, BCI paradigms which evaluate evoked potentials in the EEG follow the principles of the oddball paradigm with random sequences of target and non-target stimuli. Motivated by the goal to further increase usability and to reduce mental workload, the CharStreamer presents stimuli in a sequential order. Due to this design decision, a user is not required to be alert constantly, as it is exactly known when the desired symbol will be presented. While removing the randomness may lead to atypical and slightly less discriminative EEG features, it also introduces additional temporal structure to the ERP responses [20], which can be exploited by an adapted data analysis procedure. Therefore, novel principles for data processing and classification are introduced.

## Methods

### 2.1 Paradigm Design

**Figure 1:**
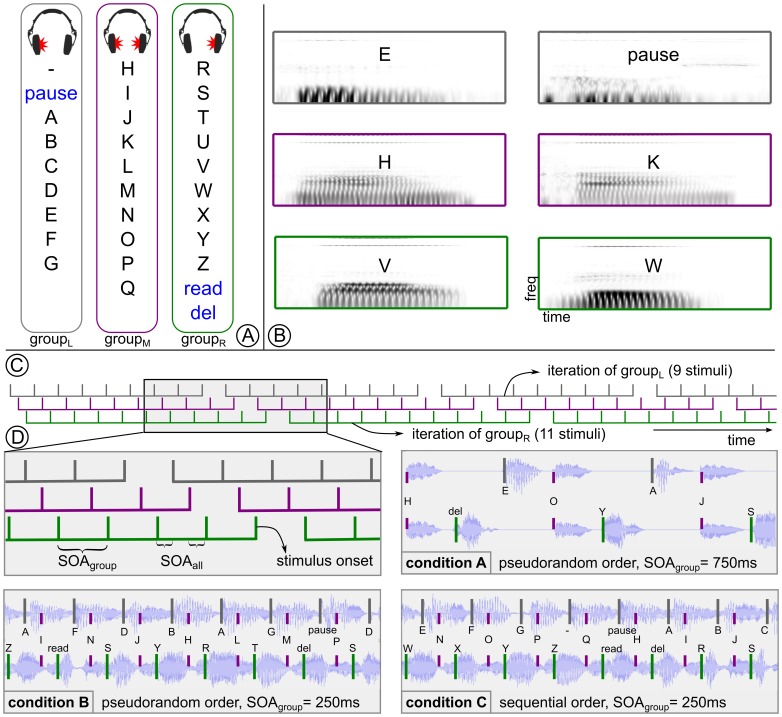
The alphabet consisting of 30 characters and symbols was split into 3 consecutive groups (A). Each group of letters is presented from a different direction. Spectrograms of six selected auditory stimuli are shown in **B**. The course of a trial is shown in **C**, depicting a sequence of several consecutive iterations. Part **D** visualizes excerpts with ∼2 seconds duration. To illustrate the mapping of the three groups to the stereo headphone tracks, the corresponding waveforms for each condition are displayed in the background. Moreover, a magnification of plot **C** is provided in the top-left corner of **D**.

The *CharStreamer* paradigm was designed such that it is very easy to understand and usable in an intuitive manner. The whole alphabet, i.e. 26 characters plus 4 command items was split into three groups, *group_L,M,R_* with *L, M, R* representing left, middle, and right respectively. The letters which were contained in one group were read out by the same voice and from the same direction (left, middle and right side). The exact division is shown in Figure 1 A. Stimuli from all three groups were alternately presented, such that every third stimulus belonged to the same group, originating from the same voice and direction. It should however be noted that the number of characters in the groups differed (9,10,11 characters in *group_L,M,R_*). Stimuli were presented in group-wise iterations, while each stimulus was presented exactly once in one iteration (see Figure 1 C). The length of one iteration varied between 9 and 11 stimuli for the three groups. Each stimulus had a duration of 200–250 ms. Although stimuli from all three groups were presented in parallel, two characters never had the exact same onset. Due to the regular temporal distribution of stimuli into the three groups, the perceived stimulus onset asynchrony (*SOA_all_*) was three times as fast as the group-wise SOA (*SOA_group_*), see Figure 1 C. The paradigm was tested in three experimental conditions (condition A–C, see Figure 1 C) with varying parametrization:

- **Condition A** is a slow oddball condition (*SOA_group_ = *750 ms, *SOA_all_ = *250 ms, pseudo-random stimulus order).
- **Condition B** is a fast oddball condition (*SOA_group_ = *250 ms, *SOA_all_ = *83.3 ms, pseudo-random stimulus order).
- **Condition C** is a fast sequential condition (*SOA_group_ = *250 ms, *SOA_all_ = *83.3 ms, fix stimulus order): the stimulation order was not random but instead following the fix order of the alphabet. Thus, the user always knew exactly when the target letter would appear.

Due to the split of the alphabet into three parts with unequal number of letters, there was no fix neighborhood of letters across groups (see Figure 1 C). For example, when F is the target letter and O and Y are the following stimuli after the first occurrence of F, then N and W will follow after the second occurrence of F.

Exemplary audio files for each condition are also published, see Audio S1–3. While condition A and B can be regarded as control conditions, condition C is finally named the “CharStreamer paradigm”, as it is the most advanced and most user-friendly setup.

### 2.2 Auditory Stimuli

The selection and optimization of stimuli for BCI paradigms based on evoked potentials is a crucial aspect. For visual paradigms, the effects of stimulus properties have been described by various authors [21]–[24]. The impact of stimulus properties has also been studied in the field of auditory BCI [25]–[27]. Moreover, polyphonic music has been recently explored as a novel stimulation approach for BCIs [28]. The authors underline the importance to carefully select and optimize stimuli. The optimization criteria are partially contradictory, as stimuli should have natural characteristics while being highly distinguishable, highly standardized and not be too arousing.

For our study, the spoken alphabet was recorded by three speakers with naturally differing voices (2 male, 1 female), and two of them with an obvious accent. The recording was processed such that an individual auditory stimulus (with a maximum duration of 250 ms) was obtained for each letter. While compressing some sounds in time became necessary, the natural characteristics of the voice, the pitch and the individual intonation was preserved as far as possible. The alphabet was recorded with German intonation and pronunciation. In order to prevent confusions, the vowel color of single letters was slightly altered, if there was another letter with a similar sound in the same group. This applies to the letters (*C, D, E* ) of the first group and (*M* and *N* ) of the second. Spectrograms of six selected auditory stimuli are shown in Figure 1 B.

### 2.3 Study Design

Ten participants were enrolled for the study with a single session of approx. 3–4 hours duration. Each participant had normal hearing and no history of neurological disease. The study was performed in accordance with the declaration of Helsinki. The study was approved by the Ethics Committee of the Charité University Hospital (number EA4/110/09) and all participants gave written consent prior to the start of their session. The study protocol consisted of a calibration phase and an online copy-spelling phase. During recordings, participants were asked to sit still and to avoid eye-movements while focusing a fixation cross. In the calibration phase, the three conditions (A–C) were applied in a block-randomized order. In each condition, 15 characters were used for calibration and the subjects had the task to mentally focus on the target letter. They were allowed to count the target occurrences, but not explicitly asked to do so as the counting was identified to be a distracting task in a pilot experiment. At the end of each trial, participants reported with a visual analog scale, how easy/hard it was to focus on the target letter.

With 14 iterations per trial, ∼ 210 target stimuli and ∼ 6100 non-target stimuli were collected for each condition and subject.

After the calibration phase, participants were asked for subjective usability ratings on a visual-analog scale for the three conditions. Furthermore they were asked, which of the conditions they would prefer to use on a daily basis, if they had to rely on the BCI system for communication.

In the second part of their session, participants performed an online copy-spelling task. It was performed exclusively in stimulus condition C. To decode target vs. non-target epochs, a classifier was trained on the calibration data of condition C, following a “standard” procedure for feature extraction and linear classification (for details, see Sections 2.4–2.6). Participants were asked to spell the sentence MIT GEDANKEN SCHREIBEN IN BERLIN, (consisting of 32 characters incl. whitespace) without error correction. In the online spelling, a dynamic stopping method was applied (for details see [29], *Höhne method* ) such that within one trial each letter was presented at least five times and maximally 12–15 times. The varying number of maximal repetitions was caused by different group sizes in *group_l,m,r_*.

### 2.4 EEG Acquisition and Preprocessing

EEG signals were recorded with a Fast’n’Easy Cap (EasyCap GmbH) using 63 monopolar, wet Ag/AgCl electrodes placed at symmetrical positions based on the extended international 10–20 system. Channels were referenced to the nose. Electrooculogram (EOG) signals were recorded via bipolarly referenced electrodes (vertical EOG: electrode Fp2 vs. an electrode directly below the right eye; horizontal EOG: F9 vs. F10). Two 32-channel amplifiers (Brain Products BrainAmp) processed the signals by an analog bandpass filter between 0.1 Hz and 250 Hz before digitalization (sampling rate 1 kHz). After applying the analog filter, the EEG raw data were first high-pass filtered at 0.2 Hz, then low-pass filtered at 25 Hz, both by a causal Chebyshev filter.

### 2.5 Artifact Correction

EEG signals are generally very prone to muscle and eye artifacts. Correcting for these artifacts was of special interest for this study, as a novel experimental paradigm is researched which might induce unknown or unexpected neural components with atypical temporal and spatial distribution. In this study, two different types of methods for artifact correction were used: a rejection method and a projection method.

To train the classifier which was applied during the original online experiment, an artifact rejection method was applied: EEG epochs violating a min–max threshold difference were rejected. This simple rejection criterion has been described in more detail in a previous study [25].

However, an offline analysis of the EEG data revealed that the above-mentioned rejection method was insufficient for the current study. Although being instructed differently, some users exhibited (unconscious) eye-movements which were partly correlated to the presentation of target stimuli. Thus, either too many target epochs were rejected (using a conservative threshold) or amplitude modulations originating from eye-movements were considered as discriminative features by the classifier when using a more liberal threshold. To circumvent both unfavorable options, an artifact projection method [30] was applied during offline analysis. This elaborate projection method automatically detects neuronal and artifactual source components derived from independent component analysis (ICA). Based on its result, artifactual components were projected out and a cleaned EEG was obtained, which was assumed to be free of eye-movement artifacts.

### 2.6 Feature Extraction and Classification

This paragraph describes the BCI data processing pipeline that was applied for the online experiment. It should be noted that only *condition C* was applied online. All target and non-target events were analyzed with a “standard” ERP processing pipeline, which is typically applied in the BBCI group for evoked potentials. This pipeline is described in detail in [31]: EEG data were band-passed filtered (0.2–25 Hz) and epoched between [−1000+1000 ms]. Artifacts were removed based on the artifact rejection method described above. Compared to other ERP-paradigms in BCI, the information contained in prestimulus EEG intervals could be considered for classification, since the user knew the stimulus order and class-discriminative EEG signals might be elicited before the stimulus onset [20]. Three to five class-discriminative time intervals were selected by a heuristic. The channel-wise mean amplitudes in those intervals were used as features. A binary linear discriminant analysis (LDA) classifier with shrinkage regularization of the covariance matrix was trained using these features.

### 2.7 Optimized Feature Extraction and Classification

**Figure 2:**
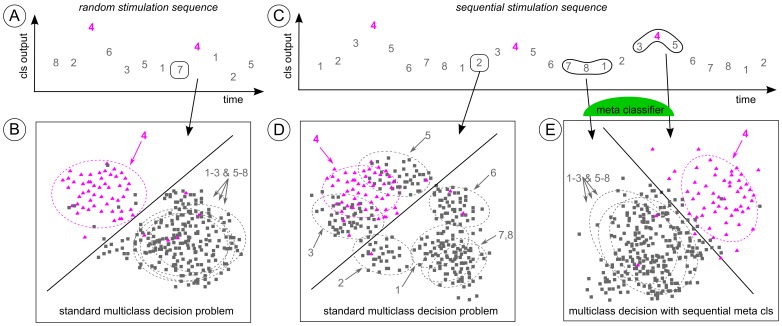
Graphical illustration of the classification problem with sequential stimuli compared to randomly ordered stimuli. The typical oddball scenario with the classification of random stimulation sequences is depicted in plot **A** and **B**. For sequential stimuli, it can be observed that classifier outputs of non-targets before or after a target behave similar to target responses (plot **C**). This leads to systematic structural distortions in the standard multi-class decision (**D**). Plot **E** depicts how a meta classifier can make explicit use of the sequential information and thereby improve the multi-class decision.

The CharStreamer paradigm (condition C) exhibits an intrinsic sequential structure. Figure 2 depicts this temporal structure and the resulting classification problem for sequential data. Therefore, the standard ERP classification procedure described above is likely to be suboptimal – as illustrated in Figure 2D.

**Figure 3:**
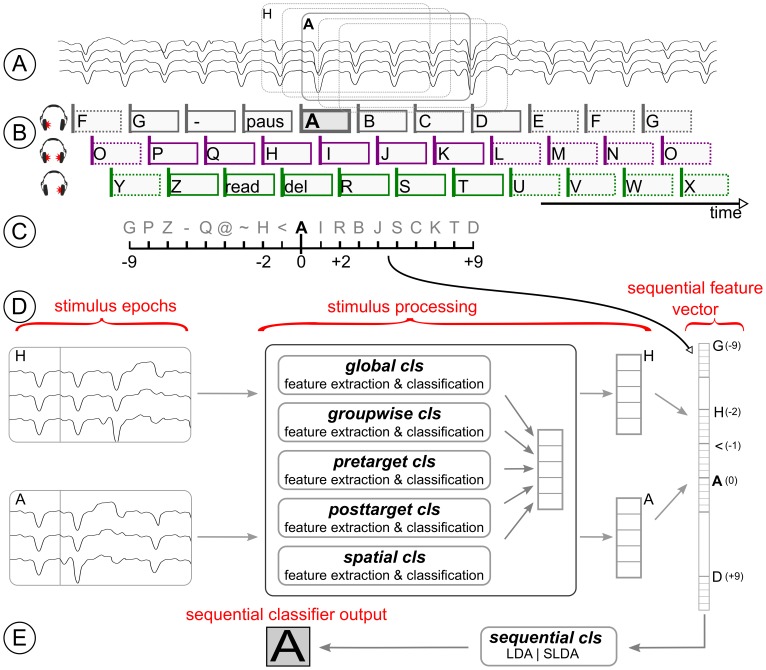
Design of the meta classifier which is optimized for sequential stimuli. Plots **A** and **B** illustrate the EEG epochs and the stimulation sequence in condition C. Plot **C** shows the range of EEG epochs which were considered in order to compute the sequential classifier output with m = 9. Plot **D** depicts the processing pipeline of stimulus epochs: each epoch was evaluated by up to five classifiers and the resulting classifier outputs were considered as features of the sequential classifier. The sequential feature vector is evaluated by a meta classier which computes a sequential classifier output for the epoch of interest (**E**).

Thus, the BCI pipeline was optimized using a meta classifier as depicted in Figure 2E. The meta classifier evaluates a sequence of outputs from several sub-classifiers. This procedure is visualized in Figure 3. Those sub-classifiers were designed in order to uncover two characteristics that were specific for the CharStreamer paradigm:

- Stimuli were presented in a sequential order with every 9th, 10th or 11th stimulus being a target. The user knew, when the next target stimulus would be presented.
- Stimuli were presented from thee directions (left, middle or right).

Each sub-classifier was calibrated with the exact same automatized procedure. The main difference between these classifiers arises from the selection of data points which were used to calibrate the respective classifier. This selection resulted in varying weights for feature extraction and classification. Given a set of training data points (EEG recording, epoched from 1000 ms before stimulus onset to 1000 ms after stimulus onset) and labels (class 1 and class 2), a “standard” binary classification approach was taken for each sub-classifier: (I) Class-discriminative time intervals were selected by a heuristic. (II) The averaged EEG data in those intervals were taken as features. (III) Classifier weights for the LDA classifier were trained with covariance shrinkage regularization [31].

The sub-classifiers are described below:

- **global cls:** the standard classification procedure was applied globally. Thus all available target stimuli and all non-target stimuli were used for calibration. This global classifier is typically used for ERP-based BCI paradigms, since it exploits high-level class-relevant information. The ratio between target and non-target stimuli in our paradigm was 1/29.
- **groupwise cls:** the standard classification procedure was applied individually for each of the three groups. This resulted in three classifiers, which were trained and applied for disjoint sets of stimuli. All target and non-target stimuli from the same group (e.g. group_L_, as shown in Fig. 3A) were used to calibrate a group-wise classifier. Thus, the classifier extracted class-relevant information (target vs. non-target) which is specific to the group. The ratio between the number of data points in class 1 and 2 was approximately 1/9.
- **pretarget cls:** the standard binary classification procedure was applied to contrast the difference between a target stimulus and its predecessor. While all available target stimuli (class 1) were taken for calibration, only those non-targets that were presented 250 ms before a target (non-targets from the same direction which preceded targets) were considered as class 2. The ratio between class 1 and 2 stimuli was 1/1.
- **posttarget cls:** the standard binary classification procedure was applied to contrast the difference between targets and their directly following non-targets. While all available targets (class 1) were taken for calibration, only those non-targets that were presented 250 ms after the target (i.e. non- targets from the same direction which followed a target) were considered as class 2. The ratio between class 1 and 2 was 1/1.
- **spatial cls:** the standard binary classification procedure was applied to exploit whether the user is attending to the left, middle or right. Thus, a binary classifier was trained for each direction/group. To calibrate each of these classifiers (e.g. for the attended left direction), all stimuli from group_L_ (targets and non-targets) were distributed into class 1 and 2. Those stimuli that were presented while the user was attending to the intended direction (e.g. left) were considered as class 1. All other stimuli which were presented while the user was attending to a different direction were considered as class 2. The ratio between class 1 and 2 was approximately 1/2 for each direction.

The meta classifier evaluated the outputs of the above mentioned sub-classifiers. In order to reduce the number of noisy features in the meta classifier, each sub-classifier had to fulfill a minimum binary classification accuracy: only those sub-classifiers featuring a binary classification accuracy of more than 65% (assessed by cross-validation on the training data) were evaluated by the meta classifier. However, the meta classifier was trained to also uncover sequential effects (see Fig. 2). The meta classifier response of the *i* th stimulus depended on the sub-classifier outputs of the stimulus sequence *i−m* to *i+m*. Thus, *m* preceding and *m* following stimuli were also considered. An example with *m* = 9 is shown in Figure 3. This design resulted in a meta classifier (called “sequential classifier” in the following) which considered up to 5 × ((2 × *m*) +1) dimensions. As model selection, the hyperparameter *m* ∈ {0,1..9}, and the classification algorithm (LDA, sparse LDA [32]) were chosen by 5-fold cross validation.

The calibration data were used to train the sequential classifier. Moreover, the resulting binary classification accuracy was assessed by nested cross validation. To assess the performance for the online experiment, the EEG data from the Copy-Spelling task was re-analyzed. Therefore, the artifact projection filter as well as the sequential classifier were trained on the calibration data only. Note that during the actual online experiment, a standard ERP classifier (see Section 2.6) was applied without the artifact projection method.

## Results

### 3.1 Usability Ratings

**Figure 4:**
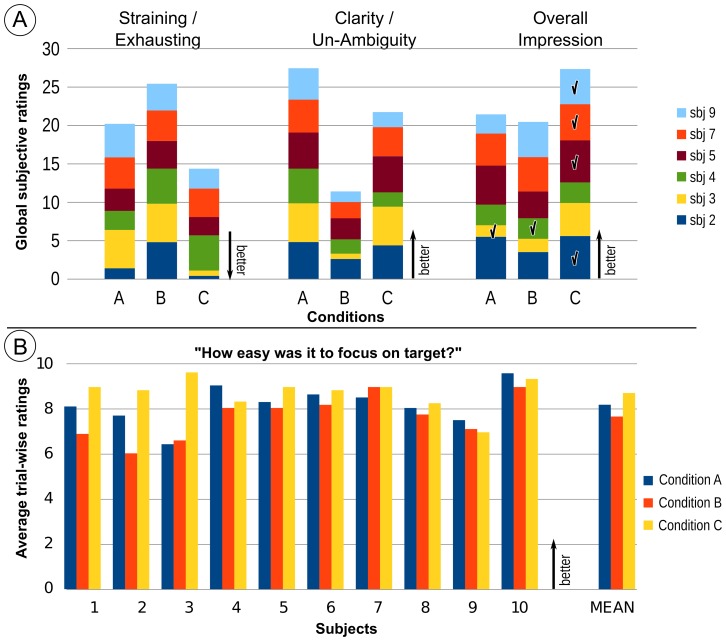
Usability ratings for the three conditions. Plot **A** shows the global subjective ratings for each condition. The overall preference for daily use is indicated for each participant by a tick mark. Arrows indicate, if larger or smaller ratings are better. Plot **B** depicts the average rating, of how well the user could focus on the target letter during each trial in the calibration.

Figure 4A depicts the behavioral ratings for the three experimental conditions, which was assessed after the calibration phase of the experiment. Note that only six out of the ten subjects are shown as the remaining four data sets were not saved due to data loss. Despite the fast stimulation speed, participants clearly rated condition C to be the preferred condition, being the least tiring condition with a clear target stimulus. This finding was supported by the average trial-wise behavioral rating (Figure 4B) which indicate, how easy it was for the user to focus on the target letter. The usability ratings thus show that condition C was the preferable condition for most subjects.

### 3.2 Physiology

**Figure 5:**
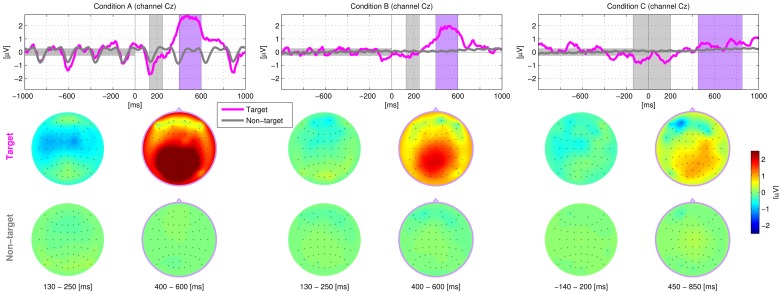
Grand averaged ERPs for conditions A, B and C. It should be noted that the stimulation speed of condition A is slower than in condition B and C.

Figure 5 shows the ERPs for each condition A, B and C averaged across all subjects. As it was expected for auditory oddball paradigms, typical N200 and P300 responses were found for conditions A and B. Due to the slower stimulation speed (SOA), both components were more discriminative in condition A than in condition B [33]. For the sequential condition C, neither the classical N200 nor the P300 component was present in the grand-average. Instead, a slower class-discriminative negativity between −200 and +200 ms was observed in the grand average. However, EEG responses of condition C showed a high variation between subjects - with multiple components having their individual temporal and spatial distributions. The ERPs of three exemplary subjects are shown in Figure S1.

### 3.3 Offline Analysis of Calibration Data

All following analyses were performed after removing artifacts caused by muscle activity and eye movements. Therefore, the artifact projection method as well as the artifact rejection method were applied as described in Section 2.5.

#### Binary accuracy

**Figure 6:**
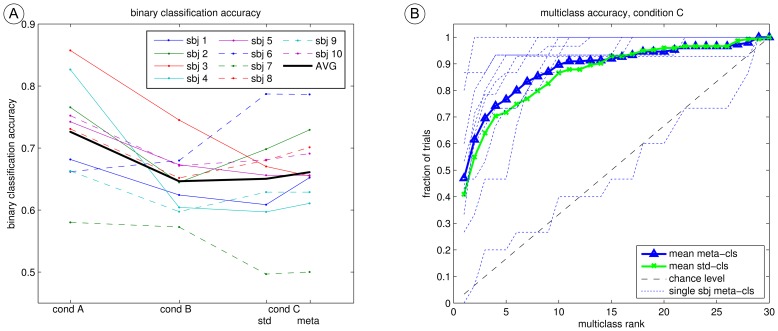
Classification accuracy for the calibration data of three conditions. The binary classification accuracy, estimated with cross validation is plotted for each condition and subject (A). The thick black line marks the mean. Plots **B** depicts the multi-class accuracy for the two classification approaches (“std” and “meta”). This was estimated by cross-validation on calibration data, using entire trials as test sets. Precisely, the point for rank = *i* quantifies the fraction of trials with a rank of the target class equal or lower than *i*. Thus, the mean multi-class performance (correct decision – rank = 1) was 47% (41%) for the meta (std) classifier. One can observe that 77% (72%) of the trials have a multi-class rank better or equal than 5. While perfect BCI control (each 30-class decision is correct) would result in a straight line with y = 1, the dashed line marks the multi-class accuracy based on chance level.

Figure 6A reveals, that condition A yields to the highest average binary accuracy. The slower timing leads to ERPs with larger amplitudes which can be classified more accurately [33]. On average, the sequential condition C elicits an equal classification accuracy compared to the oddball condition B. However, there is a high variance across subjects: For subject 3, condition B clearly outperforms condition C. Subjects 2 and 6 display the contrary behavior with condition C outperforming condition B. Moreover, the meta classifier leads to an improved classification performance compared to the standard classification approach with subjects 1 and 2 featuring an extraordinary improvement.

#### Multi-class accuracy

Due to the high number of classes (1-out-of-30 decision), the pure multi-class accuracy (i.e. fraction of correct decisions) might be a troublesome all-or-nothing metric. It doesn’t reward the situation when the true target class is identified as second-best (or third-best) class. The same holds for the ITR calculation by Wolpaw’s formular [34] as this is also based on the fraction of correct decisions. Therefore, also the rank of target class was quantified for this study. Figure 6B provides additional information in order to visualize the resulting multi-class accuracy. For all possible rank positions *r* = 1 to *r = *30 on the x-axis, the graph accumulates, how often the true class was contained within those first r ranks. Thus, the first entry on the x-axis (multiclass rank = 1) gives the “standard” multi-class performance, as it resembles the fraction of trials with a correct class decision. Accordingly, the average multi-class performance was 47% for the meta classifier and 41% for the standard classifier (chance level is 1/30 = 3.3%). However, the behavior of the graphs in 6B for ranks greater than 1 is very meaningful. It can be seen that on average, 77% (72% for the standard classifier) of the trials have a rank better than or equal to 5.

#### Class-discriminative time intervals

**Figure 7:**
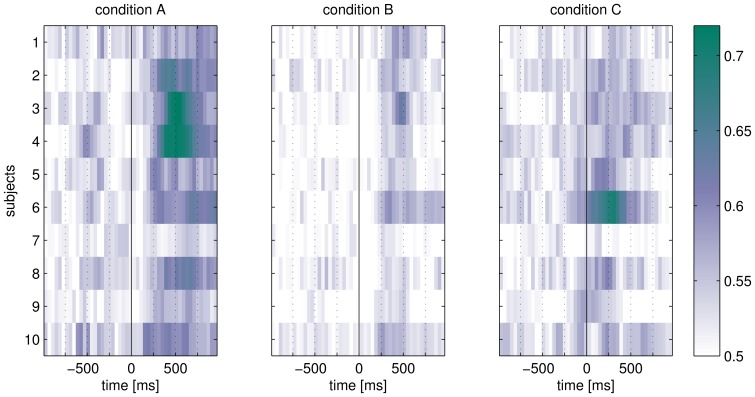
Comparison of class-discriminative information contained in the time structure of one epoch. For each subject and condition, one row depicts the estimated sliding binary classification accuracy (SBCA) of a window of 60 ms width. It was estimated by cross-validation with a 0.5 chance-level.

Figure 7 depicts discriminative time intervals for each subject and condition. It can be observed that epochs of condition A contain more discriminative features, as the estimated classification accuracy is generally higher than for the other conditions. This stands in line with the results described in Figure 6a. Condition A moreover exhibits discriminative time intervals primarily between 200 and 800 ms after stimulus onset, which corresponds to the N200 and P300 component. Compared to condition A, data from condition B has generally fewer discriminative features that are also shorter - between 250 and 600 ms after stimulus onset. As the stimulation speed is the only difference between the two conditions (condition B exhibits a three times faster stimulation speed than condition A), it can be argued that the SOA has a high impact on the discrimination of evoked potentials [33]. For condition C, discriminative EEG components are observed considerably earlier - even before the stimulus was presented. Moreover, the components are not as temporally concise as one would expect for an oddball experiment (condition B).

### 3.4 Online Spelling Accuracy

An online copy spelling with the sequential condition C was performed with nine out of ten subjects. For subject 10, there were technical problems which prevented the copy spelling run, such that online data was not recorded. A thorough reanalysis of the offline and online data revealed that the classifiers which were used during the online experiment of multiple subjects were severely distorted and partially driven by involuntary eye movements. Therefore, the results obtained during the online experiment are not shown here.

**Figure 8:**
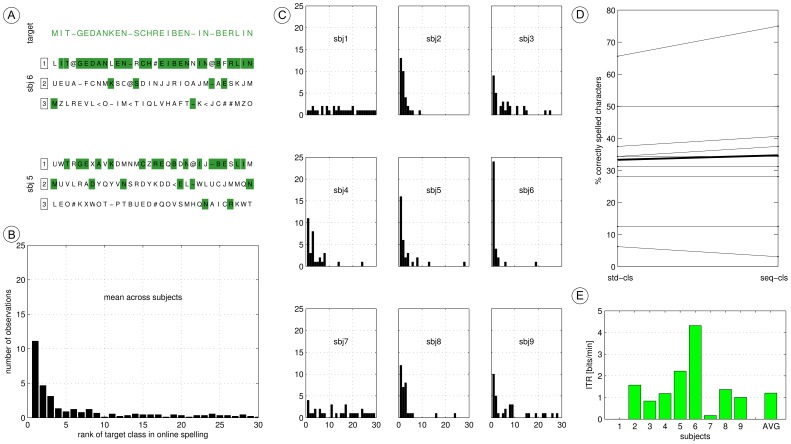
Online spelling accuracy. Plot **A–C** describe the spelling accuracy obtained by the sequential classifier. The target sentence and the top-3 ranked characters of two users are shown in **A**. Histogram **B** depicts the rank of the target letter averaged across subjects. The individual rank-histograms are shown in C. Plot **D** depicts the spelling accuracy (rank = 1) of the standard classifier and the sequential classifier for each subject and the grand average (thick line). Plot **E** depicts the information transfer rate for each subject.

However, both calibration data and online spelling data were reanalyzed (in an offline investigation after the experiment) using an ICA projection method (see Section 2.5) to filter out artifacts related to eye movements. Therefore, all projection filters and classification weights were trained solely on calibration data. Online data was evaluated only once, in order to realistically simulate an online experiment in technically plausible conditions. The resulting spelling accuracy of each subject is shown in Figure 8. It was found that seven users were able to use the CharStreamer paradigm with above-chance accuracy. Displaying the strongest class discrimination in the offline data (see Figure 7), subject 6 is also the best performing subject in the online spelling with 24/32 (75%) correctly spelled characters. Having an average of 1.5 multi-class selections per minute, subject 6 showed an information transfer rate based on Wolpaw’s formula [34] of 4.3 *bits/min*, which is highly competitive for an auditory ERP paradigm, see Figure 8E. One should however note that for the ITR calculation, only the number of correct and incorrect multi-class decisions are considered, disregarding any other information in the rank of incorrect decisions. Subject 1 and 7 failed to obtain online control. Exhibiting a very low binary classification accuracy upon calibration data (see Fig. 6), a failure of online control was expected for subject 7. For subject 1, a satisfying accuracy was observed on the calibration data, which could however not be transferred to online control. Spelling results shown in Figure 8A–C are based on the sequential classifier. Investigating the top-3 ranked letters by two well performing subjects, Figure 8A reveals that the sequential classifier has still the tendency to assign a high rank to those non-targets which follow or precede the target stimulus. However, from the 32 letters to spell, 11.1 (34.7%) were correctly chosen on average across all subjects, while 4.7 (14.6%) were second-ranked, see Figure 8B. Disregarding subjects 1 and 7 from the average, 13.5 letters (42.4%) were correctly spelled and 5.7 (17.8%) were second-ranked, which points out a considerable spelling accuracy for such a user-friendly BCI paradigm. Figure 8D depicts how the sequential classifier generally obtains either equal accuracy or an improved accuracy compared to the standard classifier on the online data. An equal behavior of both approaches could rise from the fact that the sequential classifier might use a parameterization (i.e. m = 0, weights only on the global classifier) such that it behaves equally to the standard classifier.

## Discussion

In this study, a novel auditory ERP paradigm (called “CharStreamer”) is introduced, which represents a significant step towards more user-friendly brain-computer interfaces. The CharStreamer enables enormous simplifications in terms of the user interface and the workload for the user. It is shown that complexity can be shifted from the user to the system, such that the user is exposed to the simplest and most convenient BCI setup, while the internal data processing pipeline is dealing with atypical and maybe less discriminative EEG signals. The design of the CharStreamer questions two foundations of successful ERP paradigms:

- Is a randomized stimulation order necessary to elicit class-discriminative EEG components?
- Are the “classical” N200 and P300 components indispensable to drive an ERP-based BCI system?

The CharStreamer paradigm is based on an alphabetical, sequential auditory stimulation such that the user knows when the target letter will be presented. The fast and sequential design of the CharStreamer evoked neuronal components which are significantly distinct from N200 and P300 components of oddball-based auditory ERP paradigms. A central negativity before the onset of the target stimulus was observed for most subjects. It can be speculated that this EEG component may be related to an increased alertness of the subject. Moreover, it may obey a similar neurophysiological origin to the Bereitschafspotential [35], which is know to precede a (motor) execution.

Comparing existing auditory BCI paradigms to visual paradigms, another three limits of auditory paradigms are scrutinized:

- The number of classes for auditory BCI paradigms is considerably lower than for visual paradigms. While the visual MatrixSpeller [9] as well as the rapid serial visual presentation (RSVP) speller [16] can deal with 30 classes or more, existing auditory BCI paradigms were so far limited to nine classes [36]. This limitation is mostly due to complexity, since differentiating between short auditory stimuli is more complicated and demanding than differentiating between visual stimuli. The CharStreamer paradigm tries to overcome that limitation by using 30 carefully recorded stimuli. Those stimuli are simple to recognize and easy to distinguish, as they consist of the spoken alphabet, recorded from several voices. As already mentioned, the stimulus differentiation is moreover simplified by presenting stimuli in an alphabetical order.
- Due to the reduced number of available classes, auditory ERP spellers were so far incapable of presenting the entire alphabet to the user. While several visual spellers allow a 1-step approach with the letters themselves being stimuli, auditory BCI spellers either implement a 2-step spelling system [1], [37], [38] or they combine a 1-step approach with application intelligence [36]. The letter is thus represented in a highly indirect and complicated manner. For example, in the AMUSE paradigm, the letter “L” is spelled by selecting “the second letter of the third group”, which is considerably more complicated than focusing on the “L” being highlighted on the screen. As this complex structure might be a major obstacle when applying BCI paradigms with patients in need for a communication solution, the CharStreamer is the first auditory paradigm that enables direct relation between stimulus and letter. Thus, following the principle “what you see/hear is what you get”, the user only needs to focus on the presentation of letter “L” in order to spell the letter “L”.
- The stimulation speed of ERP paradigms is a crucial aspect which directly effects neurophysiology and communication rate (such as ITR) [33]: Although visual paradigms are usually confronted with technical limits such as the frame rate of the screen, Acqualagna and colleagues (2013) [16] showed that a stimulus onset asynchrony (SOA) of 83,3 ms – corresponding to ∼12 stimuli per second – is possible. However, the fastest auditory paradigm had a SOA of 130 ms [25] – corresponding to ∼7.7 stimuli per second.
  The CharStreamer design shows that auditory paradigms are not necessarily slower that visual paradigms. By arranging the stimuli in 3 streams presented from different directions, an overall SOA of 83.3 ms –∼12 stimuli per second – was enabled, while the user was still able to identify each stimulus. With such rapid sequences of stimuli, the CharStreamer paradigm is extending the limits of stimulation speed. For future studies, it might however be beneficial to use a slower stimulation as this may further increase usability as well as ERP amplitudes and classification accuracy.

All aspects mentioned above were considered to design the most user-friendly and simple-to-use auditory ERP speller. While most aspects have been individually implemented and discussed in other studies, the CharStreamer paradigm unifies those aspects into one BCI paradigm. Serial presentation of the whole alphabet was first described in the visual RSVP speller [16], [39]. Spatially distinct stimuli for auditory ERP paradigms were proposed with the auditory AMUSE paradigm [13] and later on implemented in various other approaches [2], [36], [38]. Auditory streaming paradigms, where multiple concurrent streams are presented to the user were suggested by Hill and Colleagues (2004) [40]. It was also shown [41] that one can detect the users’ attended stream based on the analysis of evoked potentials of single trials. In order to reduce workload and to increase comfort level and BCI performance of auditory BCI paradigms, it was suggested to utilize natural stimuli instead of highly standardized artificial tones [25], [27], [42]. The first ERP paradigm with non-random order of stimulation was presented in [20].

Behavioral data showed that the chosen simplifications tremendously improve the usability of the BCI paradigm. However, such simplifications also raise the need for novel computational methods in order to establish a functioning system: it was found that the raw EEG data was contaminated with involuntary eye-movement artifacts, which had to be projected out.

Therefore, an ICA-based artifact projection method was applied in an offline analysis of both calibration and online spelling data. It should be noted that this linear projection was applied as a preprocessing step, prior to feature selection and classification. The parameters of the projection were assessed based on the calibration data only, which is essential in order to obtain a technically plausible online system. Moreover, it was observed that due to the sequential structure in the data, the classifier had problems to differentiate neighboring stimuli, thus confusing targets with their preceding or following non-targets. Therefore, a meta classifier was developed in order to improve classification accuracy for sequential ERP data. The concept of applying an meta classifier in the BCI framework is far from novel, as meta classifiers were already suggested for motor imagery [43], [44] or hybrid BCIs [45], [46]. However, the presented data illustrates that one can apply a meta classifier on ERP data, in order to account for intrinsic sequential effects in the data.

Restoring communication solutions for locked-in patients is the ultimate goal of most BCI research. Due to several reasons, paradigms which are simple to use and easy to understand are favorable when applying BCI with patients. Firstly, complicated interaction systems might be deterring and communication barriers could impede mandatory explanation steps. Secondly, patients might also be frustrated by the complexity of the BCI before even starting to use it.

The Charstreamer paradigm finally demonstrates that it is possible design such a user-friendly auditory BCI spelling system. Elaborate artifact projection methods as well as innovative classification approaches for sequential stimuli enable such a novel paradigm, which features a comfortable and intuitive usage as well as a competitive spelling speed.

## Supporting Information

Figure S1 **ERPs for all three conditions for subject 6, 8 and 10.**
(TIFF)

Audio S1 **This audiofile describes condition A in the calibration phase.** The user has the task to attend to the letter “X”.
(MP3)

Audio S2 **This audiofile describes condition B in the calibration phase.** The user has the task to attend to the letter “E”.
(MP3)

Audio S3 **This audiofile describes the CharStreamer paradigm (condition C) in the calibration phase.** The user has the task to attend to the letter “I”.
(MP3)
