# Correction: Towards User-Friendly Spelling with an Auditory Brain-Computer Interface: The CharStreamer Paradigm

**DOI:** 10.1371/journal.pone.0102630

**Published:** 2014-07-07

**Authors:** The PLOS ONE Staff

## Notice of Republication

This article was republished on June 19, 2014, due to multiple typesetting errors. The publisher apologizes for these errors. Please download the PDF again to view the corrected article. The originally published, uncorrected article and the republished, corrected article are provided here for reference.

## Supporting Information

File S1 Originally published, uncorrected article
(PDF)

File S2 Republished, corrected article
(PDF)
