# Supplementary figures and images for: Towards User-Friendly Spelling with an Auditory Brain-Computer Interface: The CharStreamer Paradigm
Source: PLoS One. 2014 Jun 2;9(6):e98322. doi: 10.1371/journal.pone.0098322 (PMC4041754; doi:10.1371/journal.pone.0098322)

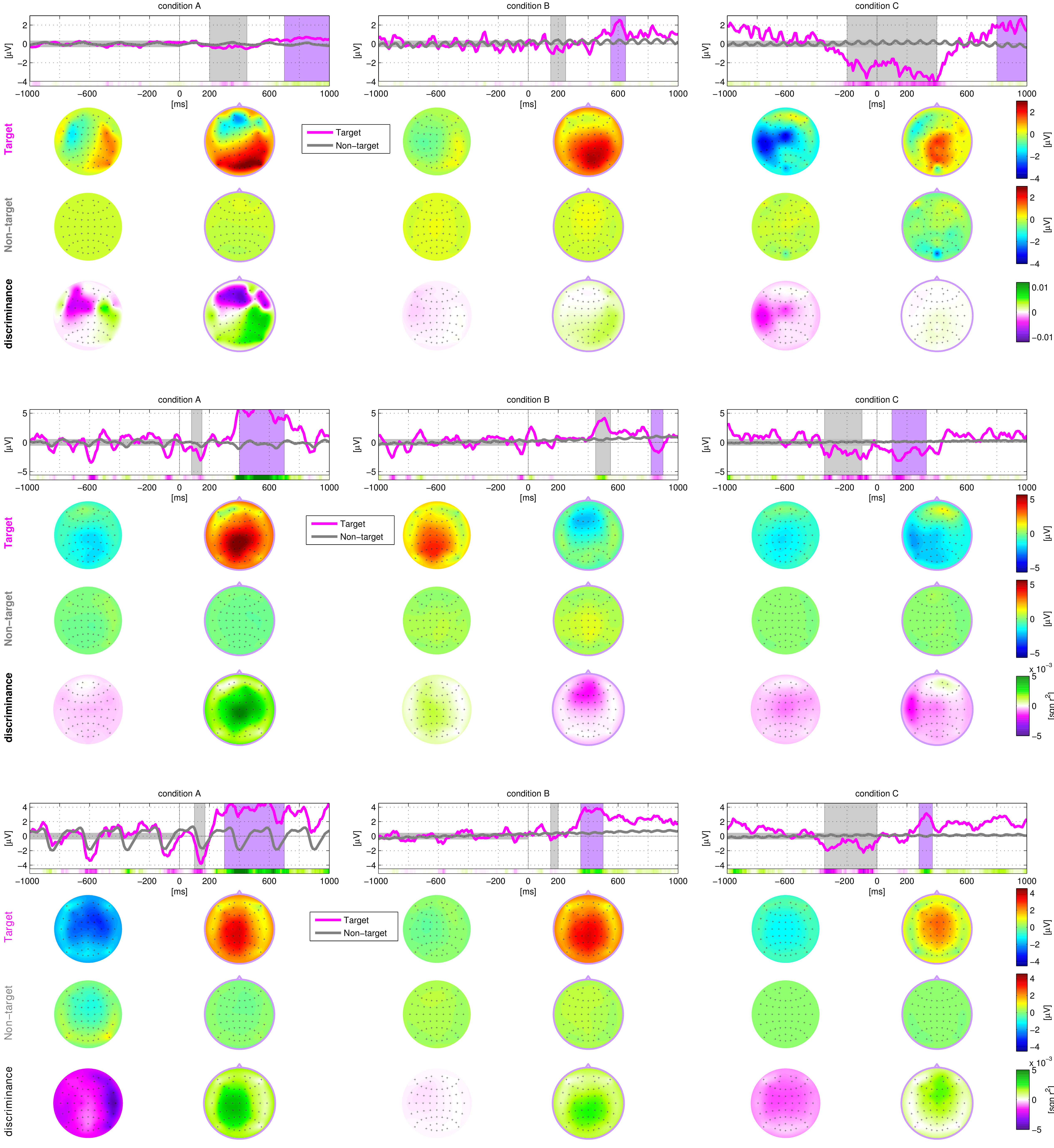

Supplement: Figure S1 — ERPs for all three conditions for subject 6, 8 and 10. (TIFF) [file pone.0098322.s001.tiff]
